# Short-Term Preoperative Calorie and Protein Restriction Is Feasible in Healthy Kidney Donors and Morbidly Obese Patients Scheduled for Surgery

**DOI:** 10.3390/nu8050306

**Published:** 2016-05-20

**Authors:** Franny Jongbloed, Ron W. F. de Bruin, René A. Klaassen, Piet Beekhof, Harry van Steeg, Frank J. M. F. Dor, Erwin van der Harst, Martijn E. T. Dollé, Jan N. M. IJzermans

**Affiliations:** 1 Laboratory of Experimental Transplantation and Intestinal Surgery (LETIS), Erasmus University Medical Center, Department of Surgery, Wytemaweg 80, 3015 CN Rotterdam, P.O. Box 2040, 3000 CA Rotterdam, The Netherlands; r.w.f.debruin@erasmusmc.nl (R.W.F.d.B.); frank.dor@imperial.nhs.uk (F.J.M.F.D.); j.ijzermans@erasmusmc.nl (J.N.M.I.); 2 Laboratory of Health Protection Research, National Institute of Public Health and the Environment, Antonie van Leeuwenhoeklaan 9, 3721 MA Bilthoven, The Netherlands; piet.beekhof@rivm.nl (P.B.); harry.van.steeg@rivm.nl (H.v.S.); martijn.dolle@rivm.nl (M.E.T.D.); 3 Department of Surgery, Maasstad Hospital, 3000 CA Rotterdam, The Netherlands; klaassenr@maasstadziekenhuis.nl (R.A.K.); harste@maasstadziekenhuis.nl (E.v.d.H.); 4 Department of Toxicogenetics, Leiden University Medical Center, Einthovenweg 20, 2333 ZC Leiden, The Netherlands

**Keywords:** dietary restriction, protein restriction, compliance, feasibility, preoperative diet

## Abstract

Introduction. Surgery-induced oxidative stress increases the risk of perioperative complications and delay in postoperative recovery. In mice, short-term preoperative dietary and protein restriction protect against oxidative stress. We investigated the feasibility of a calorie- and protein-restricted diet in two patient populations. Methods. In this pilot study, 30 live kidney donors and 38 morbidly obese patients awaiting surgery were randomized into three groups: a restricted diet group, who received a synthetic liquid diet with 30% fewer calories and 80% less protein for five consecutive days; a group who received a synthetic diet containing the daily energy requirements (DER); and a control group. Feasibility was assessed using self-reported discomfort, body weight changes, and metabolic parameters in blood samples. Results. Twenty patients (71%) complied with the restricted and 13 (65%) with the DER-diet. In total, 68% of the patients reported minor discomfort that resolved after normal eating resumed. The mean weight loss on the restricted diet was significantly greater (2.4 kg) than in the control group (0 kg, *p* = 0.002), but not in the DER-diet (1.5 kg). The restricted diet significantly reduced levels of serum urea and plasma prealbumin (PAB) and retinol binding protein (RBP). Conclusions. A short-term preoperative calorie- and protein-restricted diet is feasible in kidney donors and morbidly obese patients. Compliance is high and can be objectively measured via changes in urea, PAB, and RBP levels. These results demonstrate that this diet can be used to study the effects of dietary restriction on surgery-induced oxidative stress in a clinical setting.

## 1. Introduction

Reactive oxygen species (ROS) that the body produces during surgical procedures induce oxidative stress and lead to imbalances in homeostasis [1,2]. The subsequent stress response elicits hormonal, metabolic, and immunological changes that increase the risk of perioperative complications and may hamper postoperative recovery [1,3]. This risk is increased by preexisting factors such as obesity [4], and by perioperative factors such as ischemia-reperfusion injury (IRI) during organ transplantation [5,6]. Although treatments that decrease ROS production could reduce perioperative and postoperative complications, no effective clinical therapy is currently available.

In animal studies, dietary restriction (DR) protects against ROS-induced damage. We demonstrated previously that short-term preoperative 30% DR protects against the oxidative damage induced by renal IRI in mice and improves postoperative survival and kidney function [7,8,9], and similarly protects against liver IRI [10]. The beneficial effects of fasting on renal IRI are also observed in aged obese mice of both sexes, suggesting that DR induces protection against ROS independent of age and sex [11].

Translating DR to humans in a clinical setting is difficult, because of the effort required by patients to voluntarily restrict their calorie intake. In addition, DR goes against the generally held beliefs that patients should be well fed before surgery to prevent malnutrition. Finally, the diet composition and duration that induces similar benefits in humans as observed in rodents is not known [12]. Studies of the effects of a very low-calorie diet prior to bariatric surgery report contradictory effects on perioperative and postoperative outcomes, and adherence to the diet in these studies was not measured objectively [13,14]. In our previous pilot study in live kidney donors, we showed that three days of 30% DR followed by 24 h of fasting prior to kidney donation was feasible and safe, but had limited effects on outcome [15,16]. Subsequent results from murine experiments suggested that the beneficial effects were due mainly to restriction in protein intake [17]. The effect of protein restriction (PR) has not yet been investigated in a clinical setting.

Therefore, our current pilot study investigated the feasibility of a preoperative diet combining DR and PR in two patient populations: live kidney donors and morbidly obese patients scheduled for laparoscopic donor nephrectomy or laparoscopic bariatric surgery, respectively. To identify objective markers of diet adherence, we measured both standard and experimental metabolic markers. Our results showed that short-term DR is feasible and represents a promising next step in investigating the effects of preoperative DR on surgery-related outcome in a clinical setting.

## 2. Subjects and Methods

### 2.1. Study Design

This pilot study was designed as a prospective multicenter pilot study. The study was approved by the Medical Ethics Committee (METC, MEC number 2012-134) of the Erasmus University Medical Center, Rotterdam, The Netherlands, and by the Board of Directors of the Maasstad Hospital, Rotterdam, The Netherlands. The study procedures were in accordance with the METC guidelines. The trial is registered as the PROTECT trial in the Dutch trial registry database using trial code 3663 (www.trialregister.nl). This manuscript was prepared in accordance with the CONSORT 2010 statement [18], according to the Declaration of Helsinki.

### 2.2. Inclusion and Exclusion Criteria

The coordinating investigator approached patients at the hospitals’ outpatient clinic during their scheduled doctor appointments. All patients included in the study gave written informed consent to participate. Patients were informed that this study only aimed to establish the feasibility of the diet in surgical patients. A patient flowchart showing inclusions/exclusions and randomization procedures is depicted in Figure S1.

#### 2.2.1. Kidney Donors

Kidney donors visited the outpatient clinic at the Erasmus MC, University Medical Center Rotterdam between February 2013 and May 2014. To be eligible for the study, patients had to be between 18 and 70 years old, have a BMI ≥ 19, could not participate in another clinical trial in the 30 days prior to the day they were approached, and could have no known allergies to any of the ingredients in the diets. An additional exclusion criterion was a surgery performed outside the Erasmus MC due to participation in the cross-over kidney donation program [19]. Out of 124 kidney donors, 90 were eligible to participate in the study and were approached. Initially, 45 donors gave informed consent. After the outpatient clinic visit and before the scheduled surgery, 15 donors withdrew from the study for personal or logistical reasons (Figure S1). Included dropouts after randomization were replaced until the desired number of inclusions was reached. Thirty donors were equally (*n* = 10) randomized into each of the three intervention groups.

#### 2.2.2. Bariatric Surgery Patients

The morbidly obese patients visited the outpatient clinic at the Maasstad Hospital between March 2013 and August 2014. To participate in the study, patients had to be between 18 and 60 years of age with a BMI ≥ 40, could not have participated in another clinical study in the 30 days prior to the day they were approached, and could have no known allergies to any of the ingredients in the diets. Additional exclusion criteria were the presence of diabetes mellitus or morbid obesity caused by a known genetic syndrome or genetic defect. Diabetic patients were excluded to eliminate this confounding variable between the two surgery groups, as diabetic patients are not admitted to the live kidney donor program. Diabetes mellitus was defined as a fasted plasma glucose level ≥7 mmol/L as measured on two different days, or as either a fasted plasma glucose level ≥7 mmol/L or a non-fasted plasma glucose level ≥11.1 mmol/L with symptoms of hyperglycemia (such as thirst and polyuria). Out of 143 morbidly obese patients, 84 were eligible to participate and were therefore approached; 54 provided written informed consent. Sixteen patients dropped out of the study for various reasons (Figure S1). Included dropouts after randomization were replaced until the desired number of inclusions was reached. Since a high number of dropouts after randomization and start of the restricted occurred due to logistical reasons, additional patients were included in the restricted diet group. Eventually, 18 patients were randomized to the restricted diet, 10 patients to the DER-diet and 10 patients to the control group.

### 2.3. Dietary Intervention

All dietary interventions lasted for 5 consecutive days and were given in an outpatient setting. For the kidney donors, the diet was initiated 6 days prior to surgery. For the morbidly obese patients, the diet started between several weeks to 5 days prior to the surgery date. After providing written informed consent, patients were randomized into one of three groups. During the study, patients were offered a contact person whom they were able to approach with an accessibility of 24 h per day with questions regarding the diet, which they frequently did. Directly after completion of the diet, patients visited the outpatient clinic to evaluate their experience, and to donate a venous blood sample. The first group received a 30% DR and 80% PR restricted diet. This synthetic liquid diet containing an estimated 70% of the individual’s required calories and 20% of the individual’s protein, based on the basal metabolic rates and on the daily energy requirements (DER) as calculated with the Harris–Benedict formula [20]. The Harris–Benedict formula takes into account sex, height, age, body weight and estimated activity level. This formula is validated up to a BMI of 40. Whenever an individual had a BMI > 40, the body weight corresponding to a BMI of 40 was used to calculate the DER. Normal protein intake was set at 15% of the total calories based on the DER. Participants received calorie- and protein-restricted powder shakes (Scandishake^®^ Mix, Nutricia Advanced Medical Nutrition, The Netherlands) as the main component of the diet. The shake was provided as a powder consisting of 4% protein, 53% carbohydrates and 43% fat, and was diluted with water. The main protein source was casein with a limited amount of whey protein (Table S1). The shakes were combined with a limited amount of protein-restricted products (mainly fruits and vegetables) until the desired individual diet was reached. These protein-restricted products included: all fruits except bananas, all vegetables in a limited amount of 200 g per day with the exception of asparagus, and a maximum of one piece of gingerbread per day. The second group received a synthetic diet that was isocaloric to each individual’s DER (termed the DER-diet), which was also calculated using the Harris–Benedict formula [20]. The DER-diet was offered as a shake (Nutridrink^®^ Compact, Nutricia Advanced Medical Nutrition, The Netherlands) and was consumed without further dilution. This shake consisted of 16% protein, 49% carbohydrates and 35% fat (Table S1). A limited amount of protein-restricted products as offered to the restricted diet group, was added until the individual’s DER was reached and average protein intake was an estimated 15% of all calories. All participants, randomized to either the restricted diet or the DER-diet, were blinded to which diet they received. The third group did not receive a synthetic diet or a dietary intervention. This group continued their usual daily eating pattern. Patients were asked to keep a diet diary during the period in which patients in the other two groups received the synthetic diet. Using this diary, their daily nutritional intake was measured and calculated for 5 days, resulting in mean overall daily nutritional intake values. Experienced dieticians analyzed the diet diaries and calculated the DER, the average kilocalorie intake, and the average protein, fat and carbohydrate intake.

### 2.4. Outcome Parameters

#### 2.4.1. Subjective Measurements

To analyze subjective health outcomes, all patients were asked to fill in a standardized questionnaire, the Visual Analogue Score (VAS) for evaluation of nausea, pain and general wellbeing [21]. The VAS questionnaire uses a scale ranging from 0 to 10, with 0 representing no pain, nausea or decrease in wellbeing and 10 corresponding to the worst pain, nausea or decrease in wellbeing. The questionnaires were completed at three different time points: 1 day prior to starting the dietary intervention, on day 3 of the intervention and one day after completion of the 5-day intervention period when normal food intake was resumed. Side effects and discomfort were defined as any secondary effect related to the intervention. A distinction was made between major side effects and minor discomfort. Major side effects were defined as symptoms related to the intervention that remained days or weeks after the intervention or that required hospitalization. Minor discomfort included symptoms that caused discomfort during the intervention, but immediately disappeared after the dietary intervention.

#### 2.4.2. Objective Measurements

Before and after the dietary intervention, the following data were obtained from all patients: body weight, age, sex, length, and estimated physical activity level and duration. During the outpatient clinic visit and 1 day after the dietary intervention, two tubes of blood were collected: a 5.0 mL BD Vacutainer CPT tube (Franklin Lakes, NJ, USA) and a 5.0 mL BD Vacutainer^®^ SST™ II Advance serum tube. After centrifuging for 20 min at 1500× *g* or for 10 min at 2300× *g*, respectively, both the plasma and serum were collected and stored at −80 °C until further analysis. Only blood samples from patients that had fasted overnight were used for the analysis. “Fasted” was defined as no food intake overnight, *i.e.*, for at least 8 h prior to blood withdrawal. In serum samples the metabolic parameters albumin, urea, creatinine, glucose, ferritin, cholesterol, free fatty acids, triglycerides and high-density lipoprotein (HDL), and in plasma samples parameters prealbumin (PAB) and retinol binding protein (RBP) were measured and processed on the UniCel D×C 800 Synchron (R) Chemistry System (Beckman, Poway, CA, USA). Insulin was analyzed using the Access 2 ImmunoAssay System (Beckman) (Table S2). In addition, 143 metabolic markers were measured in serum samples by Brainshake (Brainshake Ltd., Helsinki, Finland), as summed up in a list of the service deliverables provided by Brainshake (http://www.brainshake.fi/service-deliverables-web-v1_0) [22]. A schematic overview of all of the parameters and measurements, as well as the experimental timeline, is shown in Figure S2.

### 2.5. Randomization

Randomization was performed using computer-generated lists, which were printed out and put into opaque envelopes by an employee not involved in the study. The first 30 sequential numbers of each group (*i.e.*, kidney donors and morbidly obese patients) where generated at once. Sequential numbers for the two groups were distinguishable. After these blocks, the total number of dropouts was randomized in one block by the same procedure. The coordinating investigator approached patients eligible for the study at the hospitals’ outpatient clinic during their scheduled doctor appointments. Allocation occurred after informed consent was given. All participants randomized to either the restricted diet or the DER-diet were blinded to which diet they received.

### 2.6. Statistical Analysis

Categorical data are presented as numbers (percentage) and continuous variables as mean (SD/normal distribution) or median (interquartile distance/no normal distribution). The data were tested for normality using the Shapiro–Wilks test and visual assessment. Continuous data were compared using either the non-parametric Mann-Whitney test or the *t*-test for parametric data. Related samples were analyzed using the non-parametric Wilcoxon signed rank test. Semi-quantitative scoring of the questionnaires was performed via the paired *t*-test. Significance was set at *p <* 0.05. A Bonferroni correction for multiple testing was performed on the metabolic parameters. A *p*-value of ≤ 0.002 was considered significant. The analyses were performed using Statistical Packages for Social Sciences 21.0 (SPSS Inc., Chicago, IL, USA), GraphPad Prism (GraphPad Software Inc., La Jolla, CA, USA, version 5.01), and Office Excel (2010). This study was designed as a pilot study and therefore no power calculations were performed.

## 3. Results

### 3.1. Study Population

Of the 45 live kidney donors that were included initially and who underwent randomization, 30 were equally distributed (*n* = 10) in the three groups and completed the study. Of the 54 morbidly obese patients that were included initially, 38 were distributed in the three groups with 18 patients in the restricted diet group, 10 in the DER-diet group, and 10 in the control group (Figure S3). At baseline, the morbidly obese patients group had significantly higher average body weight and BMI and was significantly more often female (Table 1). There were no differences in the baseline characteristics after randomization between dropouts and patients who completed the study.

### 3.2. Compliance with the Diets by Kidney Donors and Morbidly Obese Patients

A total of 10 live kidney donors and 18 morbidly obese patients were randomized into the restricted diet group. This group received a synthetic diet with a mean calorie restriction of 30% and a mean protein restriction of 80% relative to each patient’s DER. Twenty individuals (71%) reported completing the five-day diet (Figure S2). Eight out of 10 kidney donors (80%), and 12 out of 18 morbidly obese patients (67%) reported completing the restricted diet.

Ten donors and ten morbidly obese patients were randomized into the DER-diet. This group received a synthetic diet which resulted in a mean DR of 4% without PR. Thirteen out of 20 (65%) individuals receiving this diet reported completing the diet. Of the kidney donors, four out of 10 (40%) completed the diet; nine out of 10 (90%) morbidly obese patients completed the diet (Figure S2).

Twenty patients were randomized into the control group. Nine out of 10 donors (90%) filled in the diary for five consecutive days, while seven out of 10 (70%) morbidly obese patients filled in the diary. An analysis of the dietary diaries was performed to calculate average percentages of protein, carbohydrate and fat-intake. Nine patients in the control group had complete filled-in diaries and were included in the analysis. Average nutrient content consisted of 18% protein, 48% carbohydrates and 34% fat.

### 3.3. Discomfort during the Dietary Interventions

No major side effects were reported during or after the dietary interventions.

**Figure 1:**
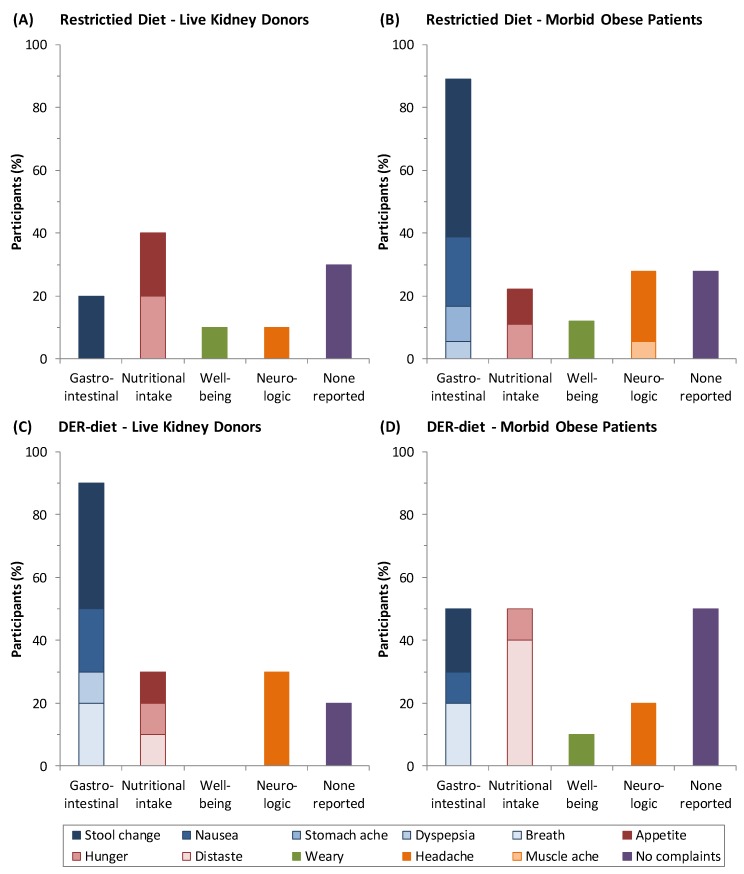
The number of side effects as percentages of participants in groups consuming a restricted diet and a daily energy requirements diet (DER-diet). (**A**) Side effects of the dietary restriction and protein restriction diet in the kidney donors were mostly related to nutritional intake and to the gastrointestinal tract; (**B**) Morbidly obese patients showed relatively more gastrointestinal discomfort; (**C**) A total of 90% of the kidney donors reported gastrointestinal discomfort during the DER-diet; (**D**) This percentage was lower in the morbidly obese patients and was the same as discomfort related to nutritional intake. Effects are clustered based on the origin of the symptoms. Within each cluster, each side effect is depicted in a different shade of color.

Twenty out of 28 individuals (71%) receiving the restricted diet reported 35 instances of minor discomfort that resolved during or directly after finishing the diet (Figure 1). In general, the Scandishake drinks were well tolerated and were reported to be palatable. A higher percentage of kidney donors (40%) than morbidly obese patients (20%) reported discomfort related to nutritional intake, e.g., hunger and appetite. In contrast, a higher percentage of morbidly obese patients (90%) than kidney donors (20%) reported gastrointestinal discomfort, e.g., stool change, nausea, stomachache and dyspepsia. Three out of eight patients whom did not complete the diet mentioned gastrointestinal discomfort as the main reason.

Of patients on the DER-diet, 13 out of 20 (65%) reported 26 instances of minor discomfort during the diet. Kidney donors reported mostly gastrointestinal discomfort (90%), while the morbidly obese reported gastrointestinal discomfort (50%) and discomfort related to nutritional intake, such as distaste and appetite (50%).

**Figure 2:**
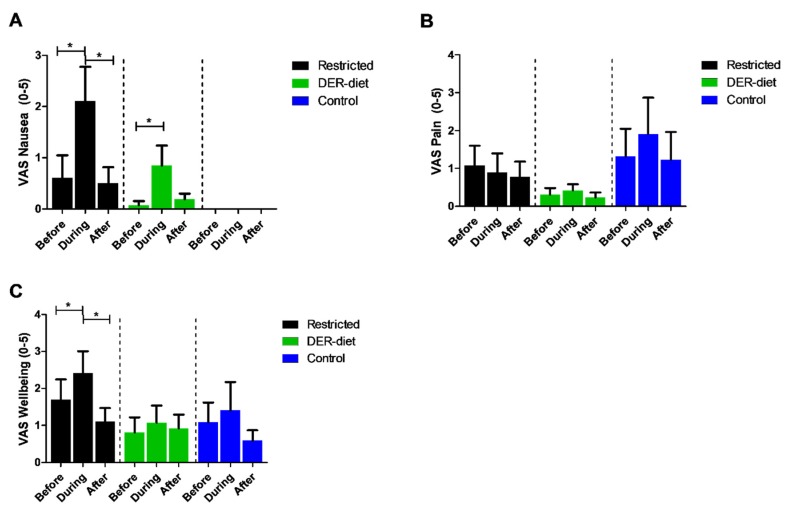
Visual Analogue Scores (VAS) for nausea, pain, and wellbeing before, during, and after each dietary intervention. (**A**) The nausea scores increased significantly for patients on the restricted diet and the DER-diet but normalized to baseline levels directly after the intervention period was over; (**B**) The pain scores did not change significantly during the dietary interventions; (**C**) The restricted diet resulted in significant decreased VAS wellbeing scores during the diet compared to before, but normalized again directly after the intervention period was over; * *p <* 0.05. Bars represent the standard error of the mean; DER = daily energy requirements.

Discomfort was scored semi-quantitatively using the VAS questionnaires at time points before, during, and after the diet. Patients that completed the restricted diet had significantly higher levels of nausea (*p* = 0.009) and decreased wellbeing (*p* = 0.02) during the diet than before the diet (Figure 2). These scores returned to baseline on day 1 after finishing the diet. There were no differences in pain scores at the three time points. Those on the DER-diet also reported higher nausea scores during the intervention (*p* = 0.04). No differences were seen for the pain scores before, during, and after the diet for either dietary intervention, no changes were reported in the control group.

### 3.4. Body Weight

**Figure 3:**
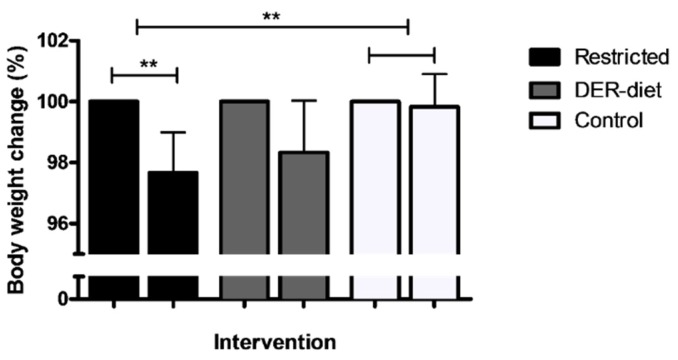
Body weight changes in the three dietary intervention groups. Patients on the restricted diet lost an average of 2.5% of their body weight, corresponding to 2.4 ± 1.4 kg; This body weight loss was significantly greater than in the control group, which showed no change; The DER-diet resulted in a 1.7% loss in body weight (1.5 ± 1.4 kg), which was not significantly different than the two other groups; Changes are shown as percentages compared to the body weight at baseline; ** *p <* 0.01. Bars represent the standard error of the mean. DER = daily energy requirements.

Individuals who adhered to the restricted diet lost on average 2.5% of their total body weight, corresponding to 2.4 ± 1.4 kg, based on the body weight measurements at the outpatient clinic before the start of the dietary restriction and on the day after its completion (Figure 3). This body weight loss was significantly greater (*p* = 0.002) than in individuals without dietary restriction (*n* = 6), who did not lose weight (0.2% of their total body weight). The body weight changes were not significantly different between the kidney donors and bariatric surgery patients. The DER-diet (*n* = 3) resulted in an average loss of 1.5 ± 1.4 kg (1.7%), which was not significantly different from either the restricted diet group or the control group.

### 3.5. Markers of Metabolism and Compliance

Before and after all dietary interventions, blood samples were collected and serum and plasma was stored for further analyses. Only samples taken from fasted patients were used for these analyses. Due to the exclusion of samples from patients who did not fast, too few samples were available from the kidney donors for statistical analysis within this group. Therefore, the data from both kidney donors and morbidly obese patients were pooled. Due to a variation in baseline levels between the different patient groups, relative differences were compared between groups using the change in values before and after the intervention.

**Figure 4:**
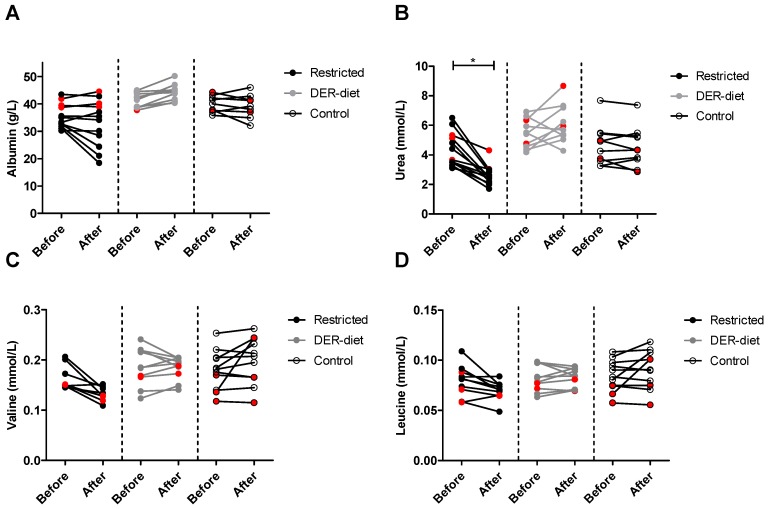
Serum levels of albumin, urea, valine and leucine after the three dietary interventions. (**A**) Serum albumin did not significantly change in any of the groups; (**B**) serum urea was decreased significantly after the restricted diet, while it did not change after the DER diet or in the control group; both serum valine (**C**) and leucine (**D**) did not differ between groups, but did show a trend towards a decrease after the restricted diet; red symbols = kidney donors, as opposed to morbidly obese individuals (black or gray symbols); DER = daily energy requirements.

Metabolic changes due to the dietary interventions as well as intragroup variations were extensively assessed via a panel of 147 metabolic parameters (Table S2). The impact of the two diets on protein metabolism was measured using serum albumin, urea and amino acids levels. The restricted diet did not significantly change serum albumin (Figure 4A). Serum albumin was increased by 7% after the DER-diet, but did not reach significance (*p* = 0.006). The increase after the DER-diet showed a trend towards higher levels compared to the relative change in the restricted diet (*p* = 0.006) and the control group (*p* = 0.003) (Table 2). No significant changes were seen in the control group. Serum urea was on average 37.5% lower after the restricted diet than before the diet (*p* = 0.002), which was also significantly different from the DER-diet (*p <* 0.001) and the control group (*p <* 0.001) (Figure 4B). There were no significant changes in serum urea in the DER-diet group or in the control group. Of the serum amino acids measured, none differed significantly between the groups (Figure 4C,D). The relative decrease of 18% in valine levels was significant compared to the relative difference of the DER-diet and the control group (Table 2). The cumulative sum of BCAAs, namely isoleucine, leucine and valine, showed a trend towards a decrease after the restricted diet (*p* = 0.005), but not after the DER-diet or in the control group. The relative decrease of 16% in the combined BCAAs also showed a trend compared to the relative change after the DER-diet (*p* = 0.004) and the control group (*p* = 0.004). Other amino acid levels were not significantly changed after any of the diets.

Neither serum markers glucose nor insulin changed significantly before *versus* after the intervention in three diet groups (Table 3). After the restricted diet, high-density-lipoprotein (HDL) was significantly decreased (*p* = 0.006), but did not remain significant after the Bonferroni correction for multiple testing. Detailed analysis of HDL-subclasses showed a trend towards a decrease in the medium HDL-particles, while other HDL-subclasses were not affected (data not shown). The DER-diet group had higher free fatty acids after the intervention (*p* = 0.01), based on an increase of saturated fatty acids (SFA) (*p* = 0.002). Furthermore, the DER-diet resulted in a significant decrease in serum cholesterol (*p* = 0.03) Lipoprotein subclasses very small very-low-density-lipoprotein (XS-VLDL), intermediate-density-lipoprotein (IDL) and large low-density-lipoprotein (L-LDL) showed a trend towards a decrease due to the DER-diet, but this did not reach significance (data not shown).

**Figure 5:**
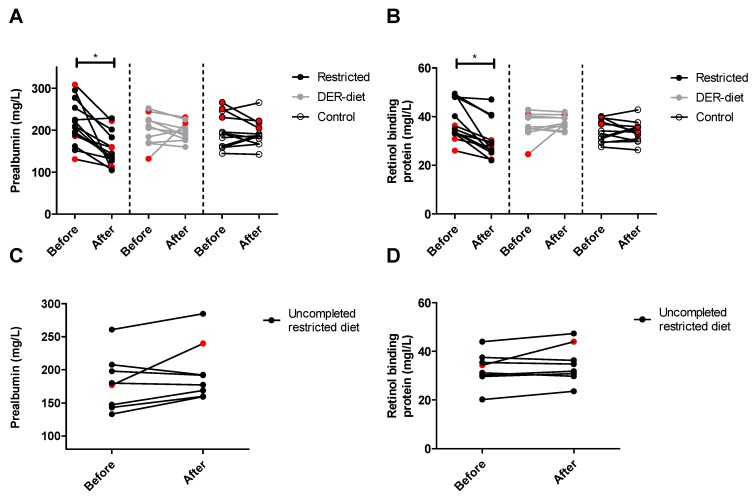
Plasma levels of prealbumin (PAB) and retinol binding protein (RBP) after the three dietary interventions. (**A**) Prealbumin (PAB) and (**B**) retinol binding protein (RBP) both decreased significantly after the restricted diet, with no changes seen in the DER-diet group or in the control group; (**C**) PAB and (**D**) RBP did not change in patients who did not complete the restricted diet. The two corresponding values for the individual patients are connected with a line. * *p* ≤ 0.002. Red symbols = kidney donors in the restricted diet group. DER = daily energy requirements.

Both plasma PAB (Figure 5A) and plasma RBP (Figure 5B) were significantly lower after the restricted diet compared to levels before starting the diet, decreasing on average 27% (*p* = 0.0002) and 22% (*p* = 0.001), respectively. All except one patient in the restricted diet group showed a decrease in serum PAB and RBP. The eight patients who did not complete the restricted diet showed an average increase of 10% in PAB (Figure 5C) and 7% in RBP (Figure 5D), which was significantly different than the measures of those who completed the diet. No significant changes were seen in the DER-diet and in the control group in both PAB (Figure 5A) and RBP (Figure 5B).

## 4. Discussion

In this study, we showed that both kidney donors and morbidly obese patients are able to adhere to a synthetic calorie- and protein-restricted diet for five consecutive days with only minor discomfort. The metabolic markers PAB and RBP showed the strongest correlation with adherence to the diet, and together with serum urea could form a potential objective marker set to validate compliance to a diet comprising 30% DR and 80% PR.

The rationale for combining DR and PR in this clinical diet was based on the known beneficial effects of short-term DR and short-term PR [17,22,23]. We showed previously that several DR regimens protect against renal IRI in mice and result in up-regulation of anti-oxidants, reduction of pro-inflammatory cytokines, improved kidney function, and increased survival [7,24]. DR also protects against IRI in aged-obese mice of both sexes, suggesting that the DR-induced effects on the stress response could be broadly applied [11].

It is not clear how the benefits of DR should be translated to humans in a clinical setting. Longer-term DR (e.g., for weeks) is considered undesirable, since feelings of hunger and fatigue and the risk of malnutrition could alter the wellbeing of patients prior to surgery. However, a shorter period could be insufficient to confer the same beneficial effects as in animal studies. Most clinical studies of preoperative DR have been performed in bariatric surgery patients and were designed to evaluate the effects of DR on weight loss and liver size reduction [13,14]. One study showed reduction in steatosis and steatohepatitis after liver resection due to preoperative dietary and fat restriction [25]. We previously used a DR regimen of three days of 30% DR followed by 24 h of fasting in live kidney donors, which proved to be feasible; unfortunately, this did not induce a beneficial response similar to that seen in mice [15,16]. This could be because the DR duration or restriction level was insufficient, or because the diet did not include PR. Based on this experience [15,16], we extended the number of days of the diet, and restricted protein intake by 80% in addition to the 30% DR.

A total adherence rate of 71% was reached, which was comparable between kidney donors and morbidly obese patients. Many factors influence adherence, such as the duration of the intervention as well as the frequency of daily doses. Osterberg *et al.* showed that the average adherence rate in clinical trials ranges between 43% and 78% [26]. They also reported an average adherence rate between 30% and 80% in patients who took three to four medication doses a day [26], which is comparable to the to three to four shakes per day in the restricted diet group of our study. In light of these results, our compliance rate is acceptable considering the fact that these patients did not receive the diet immediately prior to surgery, and therefore did not expect a beneficial effect. Further studies investigating the potential beneficial effects of this dietary regimen, might further increase the compliance rate in these patient populations.

Safety and discomfort of a preoperative diet are important factors to consider in terms of compliance and applicability. We found that serum albumin, insulin and ferritin measures did not change as a result of the restricted diet, indicating that malnutrition was not induced. As a measurement of discomfort, the VAS nausea scores were significantly increased in patients following the restricted diet, but since the scores did not exceed 2.5 points out of 10, nausea cannot be considered highly clinically relevant. The patients that withdrew early from the diet reported discomfort, mostly gastrointestinal tract-related, as the reason for withdrawal. This discomfort could be due to the liquid composition of the diet, since the change from normal food to liquid meal replacements has a direct effect on defecation. Offering patients more solid nutrition could reduce this discomfort and increase compliance. Interestingly, the morbidly obese patients more often complained about gastrointestinal symptoms during the restricted diet than the kidney donors did. A possible explanation is provided by the link between obesity and functional gastrointestinal disorders (FGIDS), such as irritable bowel syndrome and diarrhea [27]. FGIDS could make obese people more vulnerable to gastrointestinal symptoms when nutritional intake changes. During the DER-diet, both serum albumin and free saturated fatty acids increased. Together with the complaints related to gastrointestinal tract and nutritional intake, these results indicate that the patients on the DER-diet received relatively more fat than during normal food consumption. This could be the cause of nausea and stool change reported by the DER-diet groups. Based on the incidence and severity of the discomfort, together with the percentage of withdrawals from the DER-diet group and metabolic changes, we do not consider the DER-diet an appropriate control diet for future studies.

Ideally, determination of adherence to the diet would be based on objective measures. It has been shown that higher intake of calories and protein significantly increases PAB and RBP in patients at risk for malnutrition [28,29,30]. Both PAB and RBP significantly decreased in patients receiving the restricted diet in this study, while no changes were seen in the DER-diet group, in the control group, or in individuals who did not complete the diet. With only small interpatient variability, both markers therefore have great value in terms of objectively measuring compliance to a restricted diet. In addition, serum levels of BCAAs valine and leucine as well as the combination of all three BCAAs decreased in the restricted diet, with no changes in the DER-diet and the control group. This decrease with lowered protein intake is in line with a recent study by Solon-Biet *et al.* who showed that higher levels of circulating BCAAs were correlated with the percentage of protein intake [17]. Patients who received the restricted diet also showed a significant decrease in serum urea. Previous studies have shown a relationship between dietary protein intake and serum urea [31]. Interestingly, one patient provided the restricted diet showed no decrease in four of these five markers, raising doubts regarding diet compliance by this individual. Hence, a combination of these markers may very well distinguish between compliance and non-compliance to a diet comprising DR and PR. Further research is needed to validate these markers in larger cohorts of different patient populations in order to establish their independent value as compliance markers.

This pilot study has some limitations, including a high percentage of dropouts, a small sample size, and the exclusion of some blood samples that were obtained from non-fasted patients. The high number of dropouts was mostly due to logistical reasons; in some cases, the surgery date was moved up, and in others the patients did not undergo surgery. Some of the included patients declined to participate after providing written informed consent due to the stressful period prior to surgery; these patients were subsequently excluded. These logistical problems are difficult to solve, and further studies should anticipate a relatively high percentage of dropouts. Ensuring that the patients fast overnight before blood is drawn will increase the sample size and the potential value of the study. Finally, a larger sample size is needed in order to validate the results of this study and to investigate the effects of a diet comprising DR and PR on perioperative and postoperative responses.

Although safety was not an outcome measure, we have carefully monitored the patients’ peri-, and postoperative course in the present study, and have observed no differences in type and rate of complications and length of hospital stay between the three study groups.

In conclusion, our results show that a diet comprising DR and PR is feasible in both kidney donors and in morbidly obese patients awaiting surgery. This restricted diet was easily instituted, and adherence to the diet could be measured objectively using a combination of laboratory parameters. Minor adaptations to the diet, such as increasing the amount of non-liquid food during the diet, could lead to an even higher compliance rate and to decreased discomfort. This short-term dietary intervention is feasible and ready for further investigation of the effects of dietary restriction on perioperative and postoperative responses in a clinical setting.

## Figures and Tables

**Table 1:** Baseline characteristics of the study population.

Parameter	Live Kidney Donors (*n* = 30)	Morbidly Obese Patients (*n* = 38)	*p*-Value
Age, years	47 ± 13	43 ± 9	0.17
Sex, F/M	12/18	31/7	**0.003**
Body weight, kg	82.8 ± 17.3	129.1 ± 24.6	**<0.001**
BMI, kg/m^2^	26.6 ± 4.6	44.5 ± 5.4	**<0.001**

Baseline characteristics of the study population; Values are depicted as mean ± standard deviation; Significant *p*-values are depicted in bold; At baseline, the morbidly obese patients group had a significantly higher average body weight and BMI and were significantly more often female.

**Table 2:** Relative changes in metabolic parameters, amino acids and compliance markers after the dietary interventions with their corresponding *p*-value of intragroup differences.

	Intervention	Restricted Diet (% Change)	DER-Diet (% Change)	Control (% Change)	*p*-Value Restricted- DER	*p*-Value Restricted- Control	*p*-Value DER- Control
Parameter							
*Metabolic*							
Albumin (g/L)		−9	+7.2	−1.9	*0.006*	0.212	*0.003*
Urea (mmol/L)		−37.5	+13.8	−2.6	***<0.0001***	***<0.0001***	0.067
Creatinine (mmol/L)		+15.1	12.2	−2.0	0.51	*0.009*	*0.03*
Glucose (mmol/L)		−1.8	−0.6	+6.2	0.76	0.10	0.20
Ferritin		+17.5	+21.2	+20.2	0.73	0.89	0.95
Insulin (pmol/L)		+27.2	+13.7	+52.2	0.70	0.68	0.34
*Amino Acids*							
Alanine (mmol/L)		−3.6	+3.1	−5.8	0.37	0.74	0.20
Glutamine (mmol/L)		+2.1	+0.1	+2.9	0.72	0.90	0.51
Glycine (mmol/L)		+7.8	+3.3	+5.0	0.37	0.61	0.73
Histidine (mmol/L)		−11.0	−8.6	−0.2	0.72	0.15	0.12
Isoleucine (mmol/L)		−12.8	−2.7	+11.0	0.15	*0.03*	0.15
Leucine (mmol/L)		−11.1	+2.1	+8.0	*0.01*	*0.01*	0.37
Phenylalanine (mmol/L)		−4.9	+5.3	+6.4	0.06	*0.03*	0.81
Tyrosine (mmol/L)		−10.0	+5.0	0.6	0.09	0.19	0.56
Valine (mmol/L)		−17.9	+0.7	+11.6	***0.002***	*0.003*	0.22
BCAA * (mmol/L)		−16.4	−1.3%	+8.8%	*0.004*	*0.004*	0.22
*Miscellaneous*							
Prealbumin (mg/L)		−17.2	+1.0	−1.2	***0.002***	***0.0001***	0.77
Retinol Binding Protein (mg/L)		−20.5	+5.6	−0.9	***0.0002***	***0.0001***	0.26

Relative changes in metabolic parameters, amino acids and compliance markers after the dietary interventions with their corresponding *p*-value of intragroup differences. Values are depicted as mean ± standard error of the mean. Significant *p*-values are depicted in Italics. Significant *p*-values after the Bonferroni correction for multiple testing are depicted in bold and Italics. * BCAA = branched chain amino acids; cumulative sum of isoleucine, leucine and valine.

**Table 3:** Changes in metabolic parameters after the dietary intervention groups.

	Intervention	Restricted Diet			DER-Diet			Control		
Parameter		Before	After	*p*-Value	Before	After	*p*-Value	Before	After	*p*-Value
*Metabolic*										
Albumin (g/L) *		35.2 ± 1.2	32.3 ± 2.4	0.09	41.3 ± 0.8	44.2 ± 0.9	*0.006*	39.9 ± 1.0	39.0 ± 1.3	0.57
Urea (mmol/L)		4.3 ± 0.3	2.6 ± 0.2	***0.0002***	5.5 ± 0.3	6.1 ± 0.4	0.19	4.7 ± 0.4	4.5 ± 0.4	0.36
Creatinine (mmol/L)		72.6 ± 3.3	83.4 ± 4.4	***0.002***	69.5 ± 3.7	78.5 ± 5.6	*0.01*	66.4 ± 6.4	63.5 ± 5.5	0.92
Glucose (mmol/L)		4.9 ± 0.2	4.8 ± 0.2	0.53	5.7 ± 0.2	5.6 ± 0.2	0.85	5.2 ± 0.3	5.8 ± 0.3	0.13
Insulin (pmol/L) **		62.4 ± 18.6	60.9 ± 18.9	1.00	98.4 ± 18.1	106.1 ± 23.2	0.28	157.6 ± 46.3	195.5 ± 65.1	0.46
Cholesterol (mmol/L)		5.1 ± 0.5	4.5 ± 0.6	*0.03*	4.7 ± 0.3	4.5 ± 0.3	0.13	4.4 ± 0.3	4.8 ± 0.3	0.19
Free fatty acids (mmol/L)		0.52 ± 0.03	0.72 ± 0.09	0.09	0.55 ± 0.05	0.67 ± 0.04	*0.01*	0.48 ± 0.05	0.56 ± 0.09	0.49
Saturated fatty acids (mmol/L)		4.18 ± 0.25	4.01 ± 0.21	0.11	3.46 ± 0.25	3.03 ± 0.19	***0.002***	3.59 ± 0.17	3.60 ± 0.17	0.27
Triglycerides (mmol/L)		1.31 ± 0.19	1.37 ± 0.25	0.97	1.60 ± 0.42	1.51 ± 0.28	0.92	1.57 ± 0.27	1.66 ± 0.31	0.70
HDL (mmol/L)		1.32 ± 0.09	1.12 ± 0.12	*0.006*	1.27 ± 0.09	1.24 ± 0.10	0.30	1.18 ± 0.11	1.28 ± 0.11	0.59
LDL (mmol/L)		3.9 ± 0.7	4.0 ± 0.7	0.69	2.9 ± 0.3	2.7 ± 0.3	0.06	2.6 ± 0.3	2.9 ± 0.3	0.32
*Amino Acids*										
Alanine (mmol/L)		0.45 ± 0.02	0.43 ± 0.4	0.49	0.44 ± 0.02	0.45 ± 0.02	0.77	0.45 ± 0.02	0.42 ± 0.02	0.37
Glutamine (mmol/L)		0.47 ± 0.02	0.47 ± 0.02	0.63	0.49 ± 0.02	0.49 ± 0.02	1.00	0.45 ± 0.02	0.46 ± 0.02	0.35
Glycine (mmol/L)		0.26 ± 0.007	0.28 ± 0.01	0.08	0.28 ± 0.009	0.29 ± 0.01	0.85	0.25 ± 0.006	0.26 ± 0.009	0.18
Histidine (mmol/L)		0.06 ± 0.003	0.06 ± 0.004	0.08	0.07 ± 0.003	0.06 ± 0.002	*0.01*	0.07 ± 0.003	0.07 ± 0.004	0.83
Isoleucine (mmol/L)		0.06 ± 0.004	0.05 ± 0.002	0.06	0.05 ± 0.005	0.05 ± 0.003	0.43	0.06 ± 0.004	0.06 ± 0.005	0.40
Leucine (mmol/L)		0.08 ± 0.005	0.07 ± 0.003	*0.02*	0.08 ± 0.004	0.08 ± 0.003	0.63	0.08 ± 0.005	0.09 ± 0.006	0.50
Phenylalanine (mmol/L)		0.08 ± 0.005	0.08 ± 0.003	0.23	0.08 ± 0.003	0.09 ± 0.003	0.08	0.08 ± 0.004	0.09 ± 0.005	*0.03*
Tyrosine (mmol/L)		0.05 ± 0.003	0.05 ± 0.002	0.08	0.06 ± 0.003	0.06 ± 0.004	0.70	0.06 ± 0.004	0.06 ± 0.003	0.58
Valine (mmol/L)		0.16 ± 0.007	0.13 ± 0.004	*0.004*	0.19 ± 0.01	0.18 ± 0.007	1.00	0.18 ± 0.01	0.20 ± 0.01	0.17

Changes in metabolic parameters after the dietary intervention groups. Values are depicted as mean ± standard error of the mean. Significant P-values are depicted Italics, while significant *p*-values after the Bonferroni correction for multiple testing are depicted in bold and Italics; * baseline levels in the restricted diet group are significantly lower than in the DER-diet and control group. This is due to a high percentage of patients with serum albumin levels which lay below the normal values of 35–55 g/L. ** baseline levels in the control group are higher, due to patients with levels of serum insulin of >180 pmol/L.

## Supplementary Materials

The following are available online at http://www.mdpi.com/2072-6643/8/5/306/s1, Figure S1: Flowchart showing patient inclusions and exclusions, Figure S2: Timeline of the study from the first outpatient clinic visit until the end of the trial, Figure S3: Flowchart of the randomization and follow-up of the inclusions, Table S1: The composition and energy content of the restricted diet and DER-diet, Table S2: List of all parameters measured and the material and method of analysis, CONSORT 2010 checklist of information to include when reporting a randomised trial.

## Abbreviations

The following abbreviations are used in this manuscript:

DER	daily energy requirements
DR	dietary restriction
FGIDS	functional gastrointestinal disorders
IRI	ischemia-reperfusion injury
PAB	prealbumin
PR	protein restriction
RBP	retinol binding protein
ROS	reactive oxygen species
VAS	visual analogue score

## References

[1] Blackburn G.L. (2011). Metabolic considerations in management of surgical patients. Surg. Clin N. Am..

[2] Lushchak V.I. (2014). Free radicals, reactive oxygen species, oxidative stress and its classification. Chem. Biol. Interact..

[3] Kucukakin B., Gogenur I., Reiter R.J., Rosenberg J. (2009). Oxidative stress in relation to surgery: Is there a role for the antioxidant melatonin?. J. Surg. Res..

[4] Calder P.C., Ahluwalia N., Brouns F., Buetler T., Clement K., Cunningham K., Esposito K., Jonsson L.S., Kolb H., Lansink M. (2011). Dietary factors and low-grade inflammation in relation to overweight and obesity. Br. J. Nutr..

[5] Snoeijs M.G., van Heurn L.W., Buurman W.A. (2010). Biological modulation of renal ischemia-reperfusion injury. Curr. Opin. Organ Transplant..

[6] Bonventre J.V., Yang L. (2011). Cellular pathophysiology of ischemic acute kidney injury. J. Clin. Investig..

[7] Mitchell J.R., Verweij M., Brand K., van de Ven M., Goemaere N., van den Engel S., Chu T., Forrer F., Muller C., de Jong M. (2010). Short-term dietary restriction and fasting precondition against ischemia reperfusion injury in mice. Aging Cell.

[8] Van Ginhoven T.M., Huisman T.M., van den Berg J.W., Ijzermans J.N., Delhanty P.J., de Bruin R.W. (2010). Preoperative fasting induced protection against renal ischemia/reperfusion injury is independent of ghrelin in mice. Nutr. Res..

[9] Van Ginhoven T.M., van den Berg J.W., Dik W.A., Ijzermans J.N., de Bruin R.W. (2010). Preoperative fasting induces protection against renal ischemia/reperfusion injury by a corticosterone-independent mechanism. Transpl. Int..

[10] Verweij M., van Ginhoven T.M., Mitchell J.R., Sluiter W., van den Engel S., Roest H.P., Torabi E., Ijzermans J.N., Hoeijmakers J.H., de Bruin R.W. (2011). Preoperative fasting protects mice against hepatic ischemia/reperfusion injury: Mechanisms and effects on liver regeneration. Liver Transpl..

[11] Jongbloed F., de Bruin R.W., Pennings J.L., Payan-Gomez C., van den Engel S., van Oostrom C.T., de Bruin A., Hoeijmakers J.H., van Steeg H., IJzermans J.N. (2014). Preoperative fasting protects against renal ischemia-reperfusion injury in aged and overweight mice. PLoS ONE.

[12] Mitchell J.R., Beckman J.A., Nguyen L.L., Ozaki C.K. (2013). Reducing elective vascular surgery perioperative risk with brief preoperative dietary restriction. Surgery.

[13] Van Nieuwenhove Y., Dambrauskas Z., Campillo-Soto A., van Dielen F., Wiezer R., Janssen I., Kramer M., Thorell A. (2011). Preoperative very low-calorie diet and operative outcome after laparoscopic gastric bypass: A randomized multicenter study. Arch. Surg..

[14] Carbajo M.A., Castro M.J., Kleinfinger S., Gomez-Arenas S., Ortiz-Solorzano J., Wellman R., Garcia-Ianza C., Luque E. (2010). Effects of a balanced energy and high protein formula diet (vegestart complet(r)) *vs.* Low-calorie regular diet in morbid obese patients prior to bariatric surgery (laparoscopic single anastomosis gastric bypass): A prospective, double-blind randomized study. Nutr. Hosp..

[15] Van Ginhoven T.M., de Bruin R.W., Timmermans M., Mitchell J.R., Hoeijmakers J.H., IJzermans J.N. (2011). Pre-operative dietary restriction is feasible in live-kidney donors. Clin. Transplant..

[16] Van Ginhoven T.M., Dik W.A., Mitchell J.R., Smits-te Nijenhuis M.A., van Holten-Neelen C., Hooijkaas H., Hoeijmakers J.H., de Bruin R.W., IJzermans J.N. (2011). Dietary restriction modifies certain aspects of the postoperative acute phase response. J. Surg. Res..

[17] Solon-Biet S.M., McMahon A.C., Ballard J.W., Ruohonen K., Wu L.E., Cogger V.C., Warren A., Huang X., Pichaud N., Melvin R.G. (2014). The ratio of macronutrients, not caloric intake, dictates cardiometabolic health, aging, and longevity in ad libitum-fed mice. Cell Metab..

[18] Schulz K.F., Altman D.G., Moher D., Group C. (2010). Consort 2010 statement: Updated guidelines for reporting parallel group randomised trials. BMJ.

[19] De Klerk M., Kal-van Gestel J.A., Haase-Kromwijk B.J., Claas F.H., Weimar W., Living Donor Kidney Exchange Program (2011). Eight years of outcomes of the dutch living donor kidney exchange program. Clin. Transpl..

[20] Roza A.M., Shizgal H.M. (1984). The harris benedict equation reevaluated: Resting energy requirements and the body cell mass. Am. J. Clin. Nutr..

[21] Wewers M.E., Lowe N.K. (1990). A critical review of visual analogue scales in the measurement of clinical phenomena. Res. Nurs. Health.

[22] Schulz K.F., Altman D.G., Moher D., CONSORT Group (2011). CONSORT 2010 Statement: Updated guidelines for reporting parallel group randomised trials. Int. J. Surg..

[23] Harputlugil E., Hine C., Vargas D., Robertson L., Manning B.D., Mitchell J.R. (2014). The tsc complex is required for the benefits of dietary protein restriction on stress resistance in vivo. Cell Rep..

[24] Pamplona R., Barja G. (2006). Mitochondrial oxidative stress, aging and caloric restriction: The protein and methionine connection. Biochim. Biophys. Acta.

[25] Verweij M., Sluiter W., van den Engel S., Jansen E., Ijzermans J.N., de Bruin R.W. (2013). Altered mitochondrial functioning induced by preoperative fasting may underlie protection against renal ischemia/reperfusion injury. J. Cell Biochem..

[26] Reeves J.G., Suriawinata A.A., Ng D.P., Holubar S.D., Mills J.B., Barth R.J. (2013). Short-term preoperative diet modification reduces steatosis and blood loss in patients undergoing liver resection. Surgery.

[27] Osterberg L., Blaschke T. (2005). Adherence to medication. N. Engl. J. Med..

[28] Ho W., Spiegel B.M.R. (2008). The relationship between obesity and functional gastrointestinal disorders: Causation, association, or neither?. Gastroenterol. Hepatol..

[29] Bauer P., Charpentier C., Bouchet C., Nace L., Raffy F., Gaconnet N. (2000). Parenteral with enteral nutrition in the critically ill. Intensive Care Med..

[30] Tempel Z., Grandhi R., Maserati M., Panczykowski D., Ochoa J., Russavage J., Okonkwo D. (2015). Prealbumin as a serum biomarker of impaired perioperative nutritional status and risk for surgical site infection after spine surgery. J. Neurol. Surg. A Cent. Eur. Neurosurg..

[31] Addis T., Barrett E., Poo L.J., Yuen D.W. (1947). The relation between the serum urea concentration and the protein consumption of normal individuals. J. Clin. Investig..

