# Supplementary material for: Short-Term Preoperative Calorie and Protein Restriction Is Feasible in Healthy Kidney Donors and Morbidly Obese Patients Scheduled for Surgery
Source: Nutrients. 2016 May 20;8(5):306. doi: 10.3390/nu8050306 (PMC4882718; doi:10.3390/nu8050306)
Supplement: Supplementary file 1 [file nutrients-08-00306-s001.docx]

Supplementary Materials: Short-Term Preoperative Calorie and Protein Restriction Is Feasible in Healthy Kidney Donors and Morbidly Obese Patients Scheduled for Surgery

Franny Jongbloed, Ron W. F. de Bruin, René A. Klaassen, Piet Beekhof, Harry van Steeg, Frank J. M. F. Dor, Erwin van der Harst, Martijn E. T. Dollé and Jan N. M. IJzermans


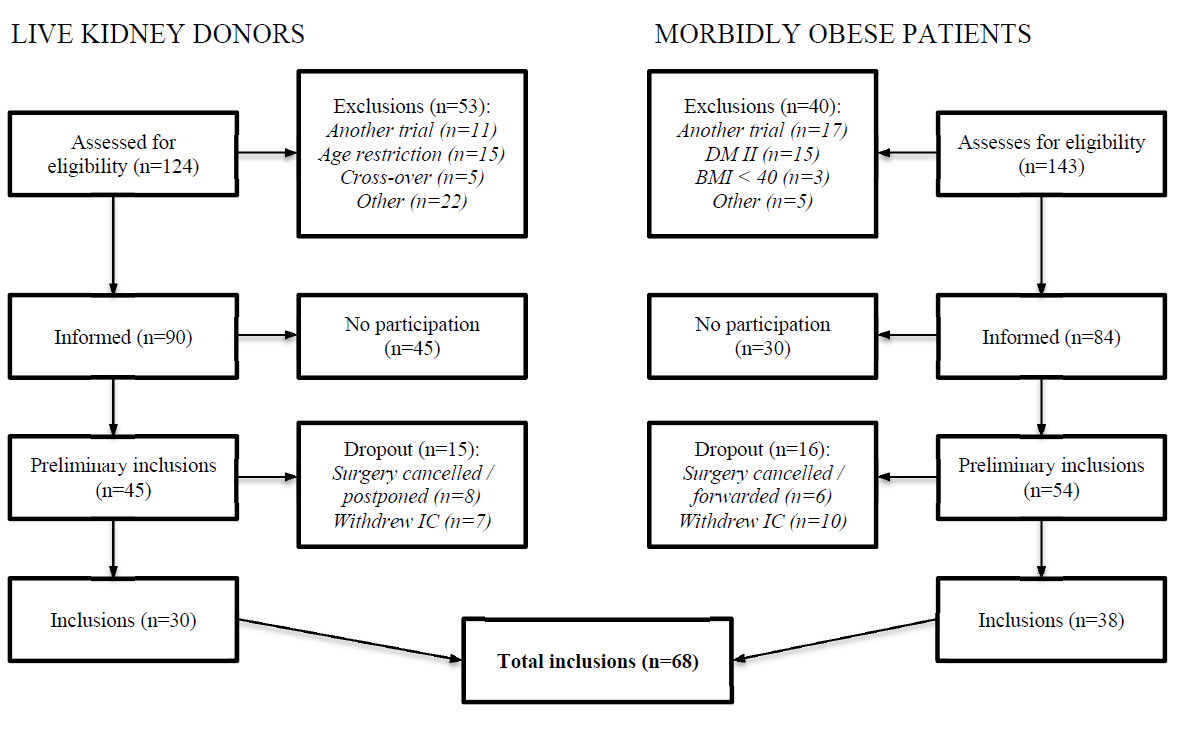


**Figure S1.** Flowchart showing patient inclusions and exclusions. Exclusions were based on preset exclusion criteria. Eligible kidney donors were approached at the outpatient clinic at the Erasmus MC, University Medical Center Rotterdam, and eligible morbidly obese patients were approached at the Maasstad Hospital, Rotterdam, The Netherlands. Additional morbidly obese patients were included after too many dropouts due to logistical reasons. BMI = body mass index. Cross-over = cross-over program at which the surgery takes place outside of the Erasmus MC, University Medical Center Rotterdam. DM II = diabetes mellitus type II. IC = informed consent.


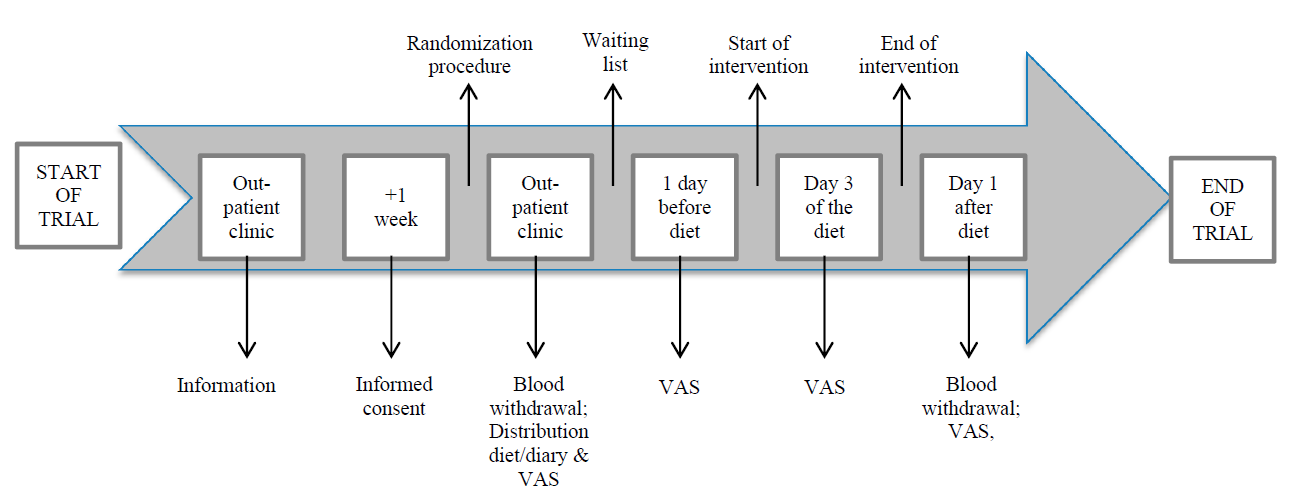


**Figure S2.** Timeline of the study from the first outpatient clinic visit until the end of the trial. Each patient was approached at the first outpatient clinic appointment, and informed consent was signed a week later. Subsequently, the randomization procedure was performed and pre-study blood samples were taken. The diets were consumed or, for the control group, the dietary diary was kept, for 5 consecutive days. The day after the diet ended, blood samples were taken again, and the trial ended. VAS = Visual Analogue Score.


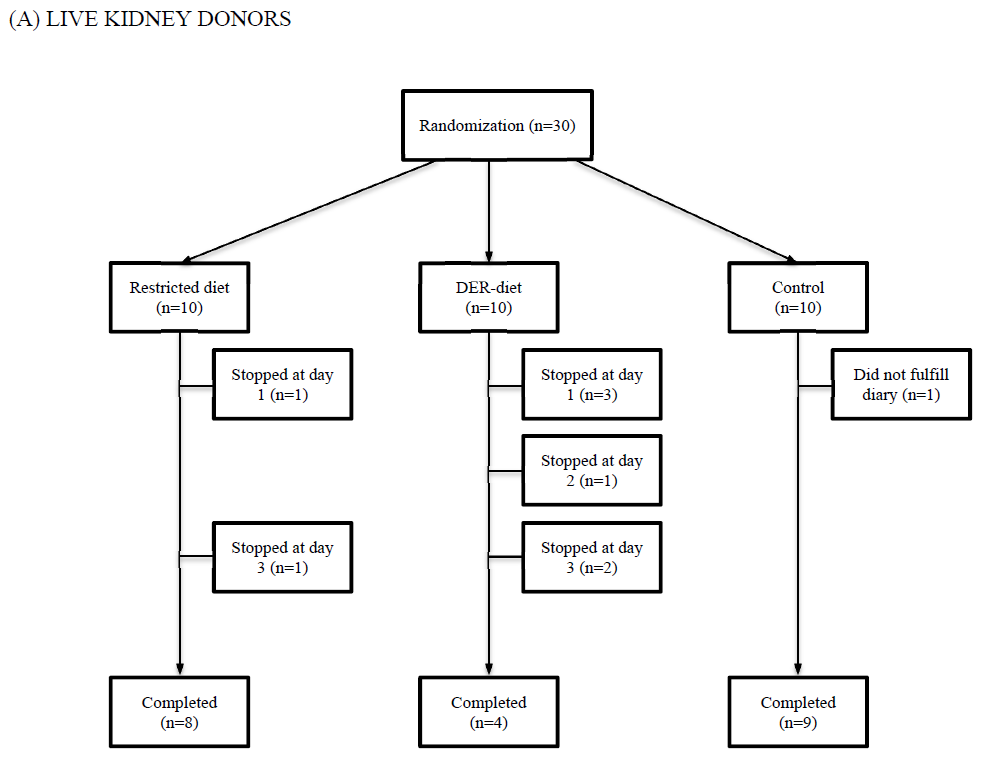

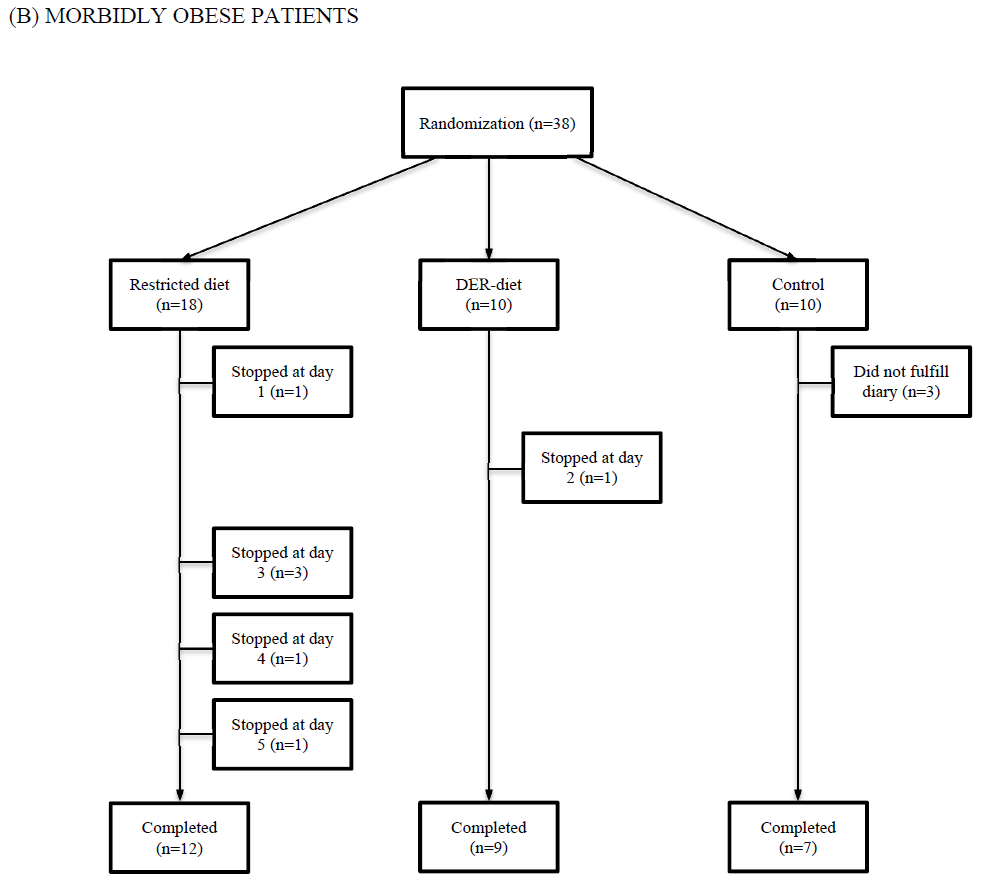


**Figure S3.** Flowchart of the randomization and follow-up of the inclusions. (**A**) Thirty kidney donors were equally randomized into each of the three intervention groups. Eight out of 10 kidney donors completed the diet; 1 stopped at day 1 and 1 at day 3. The DER-diet was completed by 4 donors; 3 stopped at day 1, 1 at day 2 and 2 at day 3. One donor did not complete the diet diary; (**B**) Twelve out of 18 morbidly obese patients completed the diet; 1 stopped at day 1, 3 at day 3, 1 at day 4 and 1 at day 5. Nine out of 10 completed the DER-diet, with only one patient who stopped at day 2. Three out of 10 patients did not complete the diet diary. DER = daily energy requirements.

**Table S1.** The composition and energy content of the restricted diet and DER-diet.

| **Diet** | **Restricted Diet** | **DER-Diet** |
| --- | --- | --- |
| Average Content per 100 gram/mL |  |  |
| Energy (kcal) | 507 | 240 |
| Energy (kJ) | 2120 | 1010 |
| Protein (g) | 4.98 | 9.6 |
| Protein (% of energy intake) | 4.0 | 16.0 |
| Casein (g) | 4.3 | 8.8 |
| Whey protein (g) | 0.4 | 0.8 |
| Carbohydrates (g) | 67.0 | 29.7 |
| Carbohydrates (% of energy intake) | 53.0 | 49.0 |
| Glucose (g) | 0.8 | 0.3 |
| Fructose (g) | 0.0 | 0.0 |
| Lactose (g) | 3.2 | <0.5 |
| Maltose (g) | 2.5 | 14.6 |
| Sucrose (g) | 14.0 | 0.0 |
| Polysaccharides (g) | 46.3 | 14.3 |
| Other (g) | 0.2 | 0.4 |
| Fat (g) | 24.5 | 9.3 |
| Fat (% of energy intake) | 43 | 35.0 |
| Saturated fat (g) | 7.7 | 0.9 |
| Monounsaturated fat (g) | 7.3 | 5.7 |
| Polyunsaturated fat (g) | 9.4 | 2.7 |
| Linoleic acid (g) | 8.1 | 2.1 |
| Α-linoleic acid (g) | 0.9 | 0.4 |
| Fibres | 0.0 | 0.0 |
| Sodium (mg) | 128 | 96 |
| Potassium (mg) | 272 | 236 |
| Chloride (mg) | 163 | 91 |
| Calcium (mg) | 85 | 174 |
| Phosphor (mg) | 147 | 174 |
| Magnesium (mg) | 30 | 33 |
| Ferritin (mg) | 0.0 | 3.8 |
| Zinc (mg) | 0.0 | 2.9 |
| Copper (mg) | 0.0 | 0.43 |
| Manganic (mg) | 0.0 | 0.8 |
| Fluoride (mg) | 0.0 | 0.2 |
| Molybdenum (µg) | 0.0 | 24.0 |
| Selenium (µg) | 0.0 | 14 |
| Chrome (µg) | 0.0 | 16 |
| Iodine (µg) | 0.0 | 32 |
| Vitamin A (µg-RE) | 0.0 | 240 |
| Carotenoids (mg) | 0.0 | 0.0 |
| Vitamin D (µg) | 0.0 | 1.8 |
| Vitamin E (mg α-TE) | 0.0 | 3.0 |
| Vitamin K (µg) | 0.0 | 13.0 |
| Thiamin (mg) | 0.0 | 0.4 |
| Riboflavin (mg) | 0.0 | 0.4 |
| Niacin (mg NE) | 0.0 | 4.3 |
| Panthotheenzuur (mg) | 0.0 | 1.3 |
| Vitamin B6 (mg) | 0.0 | 0.4 |
| Foliumzuur (µg) | 0.0 | 64 |
| B12 (µg) | 0.0 | 0.7 |
| Biotin (µg) | 0.0 | 9.6 |
| Vitamin C (mg) | 0.0 | 224 |
| Choline (mg) | 0.0 | 88 |

**Table S2.** List of all parameters measured and the material and method of analysis.

| **Biomarker** | **Abbreviation** | **Matrix** | **Method of Analysis** | **Company of Assay** | **Assay Product Number** |
| --- | --- | --- | --- | --- | --- |
| Albumin | ALB | Serum | Autoanal | Beckman | 442765 |
| Cholesterol | CHOL | Serum | Autoanal | Beckman | 467825 |
| Creatinine | CREA | Serum | Autoanal | Beckman | 442760 |
| Ferritin | FER | Serum | Immunoanal | Beckman | 33020 |
| Free fatty acids | FFA | Serum | Autoanal | Wako | NEFA-HR2 |
| Glucose | GLU | Serum | Autoanal | Beckman | 442640 |
| High-density lipoprotein | HDL | Serum | Autoanal | Beckman | 650207 |
| Insulin | INS | Serum | Immunoanal | Beckman | 33410 |
| Prealbumin | PAB | Plasma | Immunoturbi | Beckman | 475106 |
| Retinol binding protein | RBP | Plasma | Immunoturbi | Diazyme | DZ187A-K |
| Triglycerides | TG | Serum | Autoanal | Beckman | 445850 |
| Urea | UREA | Serum | Autoanal | Beckman | 442820 |

Abbreviations: Autoanal = autoanalyzer; immunoanal: immunoanalyzer; immunoturbi = immunoturbidimetry.

**Table S3.** CONSORT 2010 checklist of information to include when reporting a randomised trial *.

| Section/Topic | Item No. | Checklist Item | Reported on Page No. |
| --- | --- | --- | --- |
| Title and abstract | | | |
|  | 1 a | Identification as a randomised trial in the title | 1 |
|  | 1 b | Structured summary of trial design, methods, results, and conclusions (for specific guidance see CONSORT for abstracts) | 2 |
| Introduction | | | |
| Background and objectives | 2 a | Scientific background and explanation of rationale | 1, 2 |
|  | 2 b | Specific objectives or hypotheses | 2 |
| Methods | | | |
| Trial design | 3 a | Description of trial design (such as parallel, factorial) including allocation ratio | 2 |
|  | 3 b | Important changes to methods after trial commencement (such as eligibility criteria), with reasons | 2, 3 |
| Participants | 4 a | Eligibility criteria for participants | 2, 3 |
|  | 4 b | Settings and locations where the data were collected | 2, 3 |
| Interventions | 5 | The interventions for each group with sufficient details to allow replication, including how and when they were actually administered | 3, 4 |
| Outcomes | 6 a | Completely defined pre-specified primary and secondary outcome measures, including how and when they were assessed | 4, 5 |
|  | 6 b | Any changes to trial outcomes after the trial commenced, with reasons | N/A |
| Sample size | 7 a | How sample size was determined | 5 |
|  | 7 b | When applicable, explanation of any interim analyses and stopping guidelines | N/A |
| Randomisation: |  |  |  |
| Sequence generation | 8 a | Method used to generate the random allocation sequence | 4, 5 |
|  | 8 b | Type of randomisation; details of any restriction (such as blocking and block size) | 5 |
| Allocation concealment mechanism | 9 | Mechanism used to implement the random allocation sequence (such as sequentially numbered containers), describing any steps taken to conceal the sequence until interventions were assigned | 4, 5 |
| Implementation | 10 | Who generated the random allocation sequence, who enrolled participants, and who assigned participants to interventions | 4, 5 |
| Blinding | 11 a | If done, who was blinded after assignment to interventions (for example, participants, care providers, those assessing outcomes) and how | 4, 5 |
|  | 11 b | If relevant, description of the similarity of interventions | 4, 5 |
| Statistical methods | 12 a | Statistical methods used to compare groups for primary and secondary outcomes | 5 |
|  | 12 b | Methods for additional analyses, such as subgroup analyses and adjusted analyses | 5 |
| Results | | | |
| Participant flow (a diagram is strongly recommended) | 13 a | For each group, the numbers of participants who were randomly assigned, received intended treatment, and were analysed for the primary outcome | 5 |
|  | 13 b | For each group, losses and exclusions after randomisation, together with reasons | 5 |
| Recruitment | 14 a | Dates defining the periods of recruitment and follow-up | 2, 3 |
|  | 14 b | Why the trial ended or was stopped | N/A |
| Baseline data | 15 | A table showing baseline demographic and clinical characteristics for each group | 5 |
| Numbers analysed | 16 | For each group, number of participants (denominator) included in each analysis and whether the analysis was by original assigned groups | 5, 6 |
| Outcomes and estimation | 17 a | For each primary and secondary outcome, results for each group, and the estimated effect size and its precision (such as 95% confidence interval) | 5–12 |
|  | 17 b | For binary outcomes, presentation of both absolute and relative effect sizes is recommended | 5–12 |
| Ancillary analyses | 18 | Results of any other analyses performed, including subgroup analyses and adjusted analyses, distinguishing pre-specified from exploratory | N/A |
| Harms | 19 | All important harms or unintended effects in each group (for specific guidance see CONSORT for harms) | 6, 7 |
| Discussion | | | |
| Limitations | 20 | Trial limitations, addressing sources of potential bias, imprecision, and, if relevant, multiplicity of analyses | 14 |
| Generalisability | 21 | Generalisability (external validity, applicability) of the trial findings | 14 |
| Interpretation | 22 | Interpretation consistent with results, balancing benefits and harms, and considering other  relevant evidence | 12–14 |
| Other information | | |  |
| Registration | 23 | Registration number and name of trial registry | 2 |
| Protocol | 24 | Where the full trial protocol can be accessed, if available | N/A |
| Funding | 25 | Sources of funding and other support (such as supply of drugs), role of funders | 14 |

* We strongly recommend reading this statement in conjunction with the CONSORT 2010 Explanation and Elaboration for important clarifications on all the items. If relevant, we also recommend reading CONSORT extensions for cluster randomised trials, non-inferiority and equivalence trials, non-pharmacological treatments, herbal interventions, and pragmatic trials. Additional extensions are forthcoming: for those and for up to date references relevant to this checklist, see www.consort-statement.org.
